# Supplementary material for: Patterns of Intron Gain and Loss in Fungi
Source: PLoS Biol. 2004 Nov 30;2(12):e422. doi: 10.1371/journal.pbio.0020422 (PMC532390; doi:10.1371/journal.pbio.0020422)
Supplement: Table S1 — Also available at http://genes.mit.edu/NielsenEtAl/. (4.3 MB ZIP). [file pbio.0020422.st001.zip › NielsenEtAl/html/586.html]

AN0993.1.NCU09678.1.MG10255.1.FG04668.1


```
 CLUSTAL W (1.82) Multiple Sequence Alignments - Introns Inserted


Sequence 1: NCU09678.1	499 aa
Sequence 2: MG10255.1	509 aa
Sequence 3: FG04668.1	404 aa
Sequence 4: AN0993.1	496 aa
Alignment Length: 516 aa
Number Identitical Residues: 207 aa
Alignment Score (without introns) 11289


MG10255.1 	MSDFPASREPAVAHHGDDERPSNGDDSKTVSEDKKDHTGPPSDSDTELGEMNEKALLRKI
NCU09678.1	MAEPVTEEKSRQYSESDPDKRSVDPSSSKASET----------VDFETNSVNENSLLRKL
FG04668.1 	MNTGLKNDSPPDLSDEADTVPTEVP-------------------------VNEKALLRKL
AN0993.1  	MTDVKEIKASAPSPEREKYDIYPAEDHVAAEFT----------------DINEKSLLRKL
          	*        .    .          .    .                  .:**::****:

MG10255.1 	DWKLLPAVGILYLLSFLDRSNV1GNARIEGLLDDLGMS1GDQYLTGLTIYFVGYVIFE~I
NCU09678.1	DVRLLPAVGILYLLSFLDRSNV~GNARIEGLASDLGMT~GNQYLTGLTLYFIGYAIFE0I
FG04668.1 	DLRLLPAVGILYLLSFLDRSNV1GNARIEGMIDDLHMS1GNEYLTGLTLYFIGYVLFE0N
AN0993.1  	DYKLLPPLTILYLLSFLDRSNV1GNARLEGLTDDVGMS1GNQYLTGLTLYFVGYVLFE~I
          	* :***.: ************* ****:**: .*: *: *::******:**:**.:**  

MG10255.1 	PCNIILKRTTPQLWLPTLTVAWGIVATLMGIVTNPAGFFAARFF~LGVTESGLFPGVVYY
NCU09678.1	PCNIVLKRTTPRFWLPTLTILWGIVATLMGIVQNLTGFFIARFF~LGVAESGLFPGVVYY
FG04668.1 	----------------------------------KTGFFIARFF~LGVAESGLFP-----
AN0993.1  	PCNIILKKTTPRIWLPTLTLIWGVVATLLGIVQNYAGYLTSRTA1LGVAESGLFPGVVFY
          	...   . ::.    .: :   .  ::  .   . :*:: :*   ***:******.    

MG10255.1 	FSMWYQRRERQYRIALFFSGAALAGAFGGILAW~GIGHMRGVV~WANSWRWIFIL~EGIA
NCU09678.1	FSMWYKRRERQFRISLFFGAAALAGSFGGILAY~GIGFMRGTV~WEHGWRWIFIL~EGIA
FG04668.1 	---------------------------------~--------V1WENGWRWIFIL0EGIA
AN0993.1  	LSMWYKRNEQHYRVALFFSAASLAGAFGGILAW0GIAHMDGVG~GYAGWRWIFIL0EGLL
          	 :   .  .     :   ..:: :.: ..  :  . .   .      .******* **: 

MG10255.1 	TVAVALAAYFFIQNYPDTAKFLSPAEKRSIHARLAADSDATLDERFNWANVTAALRDPAC
NCU09678.1	TVLIAFGAYWFIYNYPDTAEFLSDKERKFIRARLASDSDATHDERFTWDNVLRAIKDPKC
FG04668.1 	TVVVAVAAYWFIENYPDTSKFLTKSERSFIHERLHADSDAIRQEKFSWAAVKEAFRDPSC
AN0993.1  	TVVMSVVAYLWVYNYPATAEFLTEKERSFITFRLKHDNDATREERFSWAAVTDAIKDPKV
          	** ::. ** :: *** *::**:  *:  *  **  *.**  :*:*.*  *  *::**  

MG10255.1 	WLYGLGFHTVSLPLYTLSLFL~PTIIANLGYTSAVAQLLTIPPYAFAFVTTLTVASLSER
NCU09678.1	WLYGLSFHTMSLPLYTFSLFL0PSIINALGYRAATAQLLTIPPYAFAFLTTLTVATVSER
FG04668.1 	WLYGLGFHTMSLPLYTLSLFL0PTIIKDLGYTAAVAQLLTIPPYAVAFVTTLCVAIASER
AN0993.1  	WLYGLGFHTMSLPLYTLSLFL0PTIIKELGYSAARAQLLTVPPYALGFVTTITVAILSER
          	*****.***:******:**** *:**  *** :* *****:****..*:**: **  ***

MG10255.1 	YRRRALPIAGSAALAIVGYVVLLANTNPVARPGVSYLGTFLAAGGIYPAVALVLSWPAIN
NCU09678.1	LHQRAIFIIGSATFAIIGYIMLLANKDPIAHPGLSYAGTFFAAGGIYPATALALSWPAIN
FG04668.1 	LAKRAVFIAGSSAVAAIGYIILLANTNPTARPGVSYVGTFFAAAGIYPGTALVLSWPAIN
AN0993.1  	TKRRAPYIMGSSAFACIGYIILLAGR----KPGVSYTGTFFCCAGIYPAVAIVLSWPANN
          	  :**  * **::.* :**::***.     :**:** ***:...****..*:.***** *

MG10255.1 	VSGQTKRAVANGMQISVGNLGAVLGTQLYRSGDGPRFIVGHSFALGYLAANIIVVLTLWR
NCU09678.1	VSGQTKRAVANAMQISIGNLGAVLGTQLYRSRDGPRYIVGHSFALGYLAGNVIVSSLLYF
FG04668.1 	VSGQTKRAIANAMQISIGNLGAVMGTQLYRSGDGPRFVVGHSMALAYLIANIVVVSILGW
AN0993.1  	VSGQTKRAIANAMQISIGNLGAVIGTQLYRTETSPRYFLGHGFSLGYLVANIIVVYILWQ
          	********:**.****:******:******:  .**:.:**.::*.** .*::*   *  

MG10255.1 	LLSRENAR------REGIAEEVKAVGALE-DWQGDTDPRWRFEV
NCU09678.1	ILSRENKK------RDGIVPEVAEVGHLK-DWDGDEDLRWRFQY
FG04668.1 	RLKKQNQS------RAAVSEEIKHVGDVE-DWKGDSDSRWRFEY
AN0993.1  	VLNRENAKKAEIRERDGLLPLMGDIGDGEGDFQGDKDPRWVFQT
          	 *.::*  .:.  .* .:   :  :*  :.*:.** * ** *:
```
